# Impact of Constant Versus Fluctuating Temperatures on the Development and Life History Parameters of *Aldrichina grahami* (Diptera: Calliphoridae)

**DOI:** 10.3390/insects10070184

**Published:** 2019-06-26

**Authors:** Wei Chen, Li Yang, Lipin Ren, Yanjie Shang, Shiwen Wang, Yadong Guo

**Affiliations:** 1 Department of Forensic Science, School of Basic Medical Sciences, Central South University, Changsha 410013, China; 2 Department of Forensic Science, School of Basic Medical Sciences, Xinjiang Medical University, Urumqi 830011, China

**Keywords:** necrophagous fly, PMI_min_, developmental duration, *Aldrichina grahami*, fluctuating temperatures

## Abstract

Necrophagous fly species are commonly used as forensic tools to estimate the minimum postmortem interval (PMI_min_). Many researchers raised necrophagous flies under constant temperature regimes to collect their developmental data. However, in most forensic cases, the ambient temperature fluctuates. In order to investigate a forensically important fly developmental mode (the Isomegalen diagram, Isomorphen diagram and Thermal summation models) and make comparisons of the developmental rate between constant temperatures and fluctuating temperatures, we used *Aldrichina grahami* (Diptera: Calliphoridae) to investigate the life history parameters at eight constant temperatures ranging from 8 to 36 °C. We also compared developmental rate of *A. grahami* in three groups of constant versus fluctuating temperatures: 8 °C vs. 6–12 °C, 12 °C vs. 10–16 °C, and 16 °C vs. 14–20 °C. Our data showed that *A. grahami* is cold tolerant with a mean (±SE) development threshold temperature (*D*_0_) of 3.41 ± 0.48 °C and a thermal summation constant (*K*) of 8125.2 ± 288.4-degree hours. The three groups subjected to fluctuating temperatures took longer to develop compared to those developing in constant temperatures when simulated in a model. These results not only provide detailed developmental data for the use of *A. grahami* in the estimation of the PMI, but also indicate that ambient temperature fluctuation must be taken into consideration for the use of all necrophagous fly species.

## 1. Introduction

Forensic entomology is the science of collecting and analyzing insect evidence to aid in forensic investigations [1]. Forensic entomologists can use developmental data of sarcophagus insects to determine the minimum post mortem interval (PMI_min_) or the time since insect colonization of human remains. Forensic investigators link the information of a crime scene or discovery location to the duration of the forensically important insects found on, in or around corpses [2,3]. Blow flies (Diptera: Calliphoridae) are one of the most important families in forensic entomology, and have a cosmopolitan distribution. With a remarkable flying capability and a striking sense of smell, they can locate and reach a corpse in a very short time after death occurs [4,5]. Blow flies are often the first insects colonizing a carcass, making them the most frequently and effectively available insects for PMI_min_ estimation [1,3].

*Aldrichina grahami* (Aldrich 1930, Diptera: Calliphoridae) is a forensically important species that is mainly distributed in Asia, but it has also been reported to appear in Russia, Mexico and the United States of America [6,7,8,9]. Adults of *A. grahami* rarely aggregate indoors, and commonly colonize garbage, feces and carcasses [9]. When the ambient temperatures are low (usually from late autumn to the following spring), *A. grahami* is one of the first species to colonize a corpse, which suggests that this species is relatively cold tolerant [9,10]. Calculation of the PMI_min_ requires the gathering of exact developmental data [3,11]. Hence, we used *A. grahami* to study the developmental models at both constant temperatures and fluctuating temperatures.

Accurate developmental data of sarcophagus flies are vital for estimation of PMI_min_. Most experiments on the developmental patterns of forensically important insects under constant temperatures aim to collect data on changes in the length of larval body, developmental stage, and thermal summation [12,13,14,15,16,17,18]. Only a few studies refer to fluctuating temperatures [15,19,20,21,22]. However, in most medicolegal investigations, the ambient temperatures of the scene are variable. As such, we raised sarcophagus flies in the laboratory by imitating the fluctuating temperatures of a scene, using temperature models of climatic conditions naturally occurring in Hunan province (28°12′ N, 112°58′ E), south-central China, in the most active seasons for *A. grahami*.

In this study, the adults of *A. grahami* were trapped in the wild to establish a laboratory colony. Collecting developmental data at eight constant temperatures of 8 °C, 12 °C, 16 °C, 20 °C, 24 °C, 28 °C, 32 °C and 36 °C, and three fluctuating temperatures of 6–12 °C, 10–16 °C, 14–20 °C, (average temperatures of three fluctuating temperatures groups were 8 °C, 12 °C, 16 °C). The body length and weight of the larvae varied with accumulated temperature, and developmental durations were used to establish relevant developmental models (the Isomegalen diagram, Isomorphen diagram and Thermal summation models). In addition, we compared the development of *A.grahami* between fluctuating temperatures and constant temperatures. All these results of *A. grahami* determined the post-colonization interval and play an important role in the estimation of the PMI_min_.

## 2. Materials and Methods

### 2.1. Establishment of Laboratory Colony

During the season of *A. grahami* exuberant reproduction in March 2016, in Changsha, China (28°12′ N, 112°58′ E), *A. grahami* adults were collected from a deserted yard near a reservoir, which was used to discard devalued fish by fishermen. Fifty adult flies were obtained. The fly species identification was performed using the adult identification key according to Fan [23]. The rearing was conducted in an insect laboratory; the temperature of the laboratory was approximately 24 °C, with 70% relative humidity and natural light. Moreover, other insects were prevented from entering this room. Adults were reared in an insect cage (35 by 35 by 35 cm) with the six sides covered by a nylon net using a zipper to conduct switches. A nest feeder and two culture dishes were placed in the cage. The nest feeder was loaded with fresh water *ad libitum*, and the water channel was covered by a plastic net to serve as a stand when the adult flies drinking. A mixture of sugar and milk powder (1:1) contained in a dish (12 cm in diameter) was used as the food, and 15 g fresh pig lung in another dish was used to induce oviposition. The eggs were reared in a flat-bottomed bowl (18 cm in diameter, 5 cm in height), in a box (25 by 25 by 12 cm) filled with 2–3 cm height of sifted sand to cover the bottom of the box, and placed the box in another insect cage (we called the combination of dish, bowl, box, and cage the biomimetic appliance for rearing larvae). As such, a sufficient amount of fresh pig lung was supplemented at every inspection every day until pupation. To obtain a pure-bred colony for the subsequent study, the flies were cultured for five generations.

### 2.2. Experiment Condition

We designed the fluctuating temperature models on the basis of the weather conditions in southeast China (http://www.tianqihoubao.com/lishi/changsha.html). For instance, temperatures of 14–20 °C (Fluctuating 16 °C = F16 °C, Constant 16 °C = T16 °C) were used, with the average temperature being 16 °C to imitate weather in April. Temperatures were designed as follows:

- 00:00–06:00 14 °C,
- 06:00–14:00 temperature increasing arithmetically from 14 to 20 °C,
- 14:00–22:00 temperature decreasing arithmetically from 20 to 14 °C,
- 22:00–00:00 14 °C.

**Figure 1:**
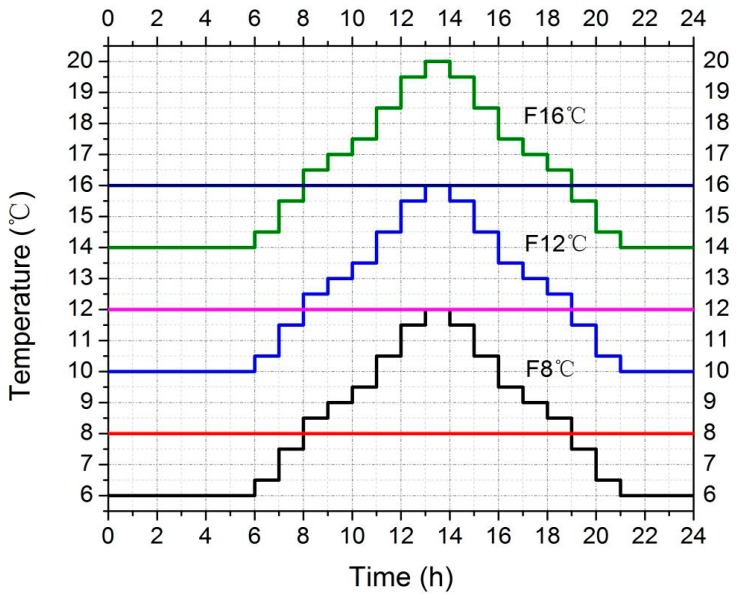
Fluctuating temperatures models.

The second condition temperatures were from 10 to 16 °C (Fluctuating 12 °C = F12 °C, Constant 12 °C = T12 °C), with average temperatures of 12 °C used to imitate weather in March and November. The third condition temperatures were from 6 to 12 °C (Fluctuating 8 °C = F8 °C, Constant 8 °C = T8 °C), with the average temperatures of 8 °C used to imitate weather in December and February. Detailed information of the three fluctuating temperature conditions is shown in Figure 1. Fluctuating temperature models were designed according to two points: 1, climatic conditions naturally occurring in the Hunan province of China; 2, we simplified the model and increased the convenience of conducting the relevant calculations.

To ensure a sufficient number of eggs, over 1000 adult flies were raised in two big insect cages (100 cm by 100 cm by 100 cm) in the insect room, in the stable oviposition period. No pig lung was provided for one day or more to make an artificial peak oviposition period. A dish containing 20 g of fresh pig lung was placed in the insect cage to induce oviposition, we collected the eggs oviposited after 2 h [24], and recorded the time of oviposition. Egg masses were made containing about 2800 eggs (regarded as a replication), then the eggs were equally divided into two portions, with one portion for constant temperatures and another portion for fluctuating temperatures. To avoid the larvae overcrowding, which would increase the temperature and stimulate the growth of larvae [1], a larger flat-bottomed bowl (22 cm in diameter, 5 cm in height) was used (we would have added another bowl if larvae were overcrowding).

Each portion and 20 g of pig lung were transferred into the biomimetic appliance for rearing larvae, and the biomimetic appliance was placed in a climatic chamber LRH-250-GSI (Taihong Co., Ltd., Shaoguan, China) at constant temperatures of 16 °C. The other portions were placed in the other climatic chamber at fluctuating temperatures of 14–20 °C (each incubators of fluctuating temperatures vs constant temperatures provided same quantity of heat in every 24 h). All climatic chambers had a 75 ± 5% humidity and a photoperiod of L12:D12. Specifically, the temperature was controlled by an electronic system to ensure that the incubators maintained the preset temperatures. In addition, the temperature and humidity of the chambers was monitored by automatic thermohygrograph (FLIR SSN-22 USA). The above experiment was repeated five times in different incubators (each replication put in a separate incubator). The remainder of the two groups of constant temperatures and fluctuating temperatures (12 °C vs. 10–16 °C, 8 °C vs. 6–12 °C) were conducted with the same processes as 16 °C vs. 14–20 °C. In addition, the group of 8 °C vs. 6–12 °C was sampled only once a day. The remaining constant temperatures (20 °C, 24 °C, 28 °C, 32 °C, 36 °C) also followed the procedures as above, with egg masses containing about 1400 eggs as a replication. For each temperature, the experiment was repeated five times.

### 2.3. Collection of Developmental Data

After oviposition, the eggs were checked hourly and the time of hatching was recorded. In addition, a small piece of fresh pig lung was provided uniformly in the culture bowl to avoid sharp accumulation of larvae-generated heat [25]. A sufficient amount of fresh pig lung was supplemented every eight hours (more frequently in high temperatures) until pupation. The supplement had to meet the requirements of the larvae for development.

The onset of the 1st instar (equivalently, the endpoint of the egg stage) was designated when 50% of the larvae hatched [25]. Ten of the larvae were sampled randomly every 12 h after hatching (more frequently in high temperatures) and immediately placed in >80 °C hot water for 30 s, then preserved in 75% ethanol with the volume doubling that of the larvae at 6 °C for no more than two weeks [11,26,27]. The length of each individual was measured using a digital caliper (Meinaite, Shanghai, China). The larvae were weighed by analytical balance (FA1204B, Shanghai, China). The weight of the larvae had a large variation even for the same cohort. Hence, we just measured the body weight of ten larvae as a single unit. Using a stereomicroscope (Motic SMZ-168) the larval instar was determined according to the number of slits in the posterior spiracle [28]. Moreover, any individual proceeded to the next stage was considered to be at the endpoint of the current stage (i.e., a 1–2 transitional instar was counted as a first-instar larva) [29]. The onset of the pupal stage was designated as the time when 50% of individuals had pupated. The same method was used to determine the wandering period and eclosion. At pupation, pupae were observed every 12 h.

### 2.4. Data Analysis

Data analysis was performed by the software OriginPro 9.0. One-way ANOVA tested the effect of temperature treatment on the duration of each life stage. The relationship between the larval body length and time after hatching was analyzed using nonlinear regression. The larval body length was used as the independent variable while time after hatching was the dependent variable [25]. The revised regression model proposed by Ikemoto and Takai was used [30]. A linear analysis was conducted to analyze the relationship between developmental duration and accumulated degree hours (ADH) at each developmental stage and the total developmental stages. The slope and y-intercept of the linear regression equation were used to determine the development threshold temperature D_0_ and thermal summation constant *K*, respectively.

## 3. Results

### 3.1. Constant Temperatures vs. Fluctuating Temperatures

For *A. grahami* reared in three groups of constant vs. fluctuating temperatures, the durations of every developmental stage decreased with increasing temperature, including the egg, first instar (1st instar), second instar (2nd instar) and third instar (3rd instar) larvae, wandering and pupae (Table 1). In addition, for each fluctuating group, the time of every developmental stage was longer than the matched constant group, and the significant difference became indistinctive with increased temperatures. Each developmental duration from 1st instar larvae to wandering in F8 °C and F12 °C was significantly longer than in T8 °C and T12 °C (Table 1, one-way ANOVA + LSD test at *p* < 0.05). Notably, the developmental durations of eggs only showed significant differences in 8 °C vs. 6–12 °C. The significant difference was indicated at pupal stage in 16 °C vs. 14–20 °C, and the adults failed to eclose (dead pupa) in the groups of T8 °C, F8 °C and F12 °C (Table 1).

**Figure 2:**
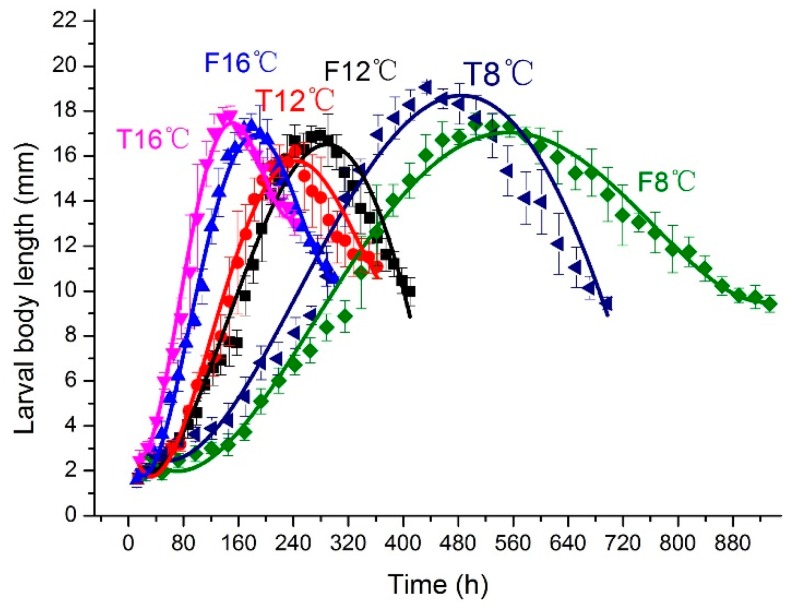
Changes in *A. grahami* larval body length (mm) over time (h) at different constant temperatures and. fluctuating temperatures. The vertical bars represent the standard deviation. The curve represents the fitting of larval body length and time.

Developmental rate (*v*) is calculated from developmental duration (*h*), *v* = 1/*h*. Because each fluctuating group had a longer time than the matched constant group in every developmental stage, the developmental rate of fluctuating temperatures was slower than constant temperatures over the whole developmental cycle. We used the time after hatching as the independent variable and larval body length as the dependent variable to obtain the curve fitting equation (Figure 2) and equations (Table 2) of the changes in larval body length of *A. grahami*. Accordingly, we tested the change in larval body weight with time (Supplementary Materials Figure S1 and Table S2). The larval body weight was connected to larval body length, thus, the rate of weight increase in fluctuating temperatures was slower than in constant temperatures.

### 3.2. Development in Constant Temperatures

#### 3.2.1. Developmental Duration and Isomorphen Diagram

From constant 12 to 28 °C, the developmental durations of the egg, 1st instar, 2nd instar and 3rd instar larvae, wandering and pupa were decreased. At 36 °C the egg of *A. grahami* was unable to hatch, and at 8 °C the adults failed to eclose. Besides, two misshapen developmental durations were observed in 3rd instar larvae and were wandering at 32 °C; both developmental durations were unnaturally prolonged (Table 1). The average time of hatching was decreased from 99.3 h at 8 °C to 14.9 h at 32 °C, and the total developmental duration was decreased from 951.6 h at 12 °C to 338.4 h at 28 °C (Table 1).

**Figure 3:**
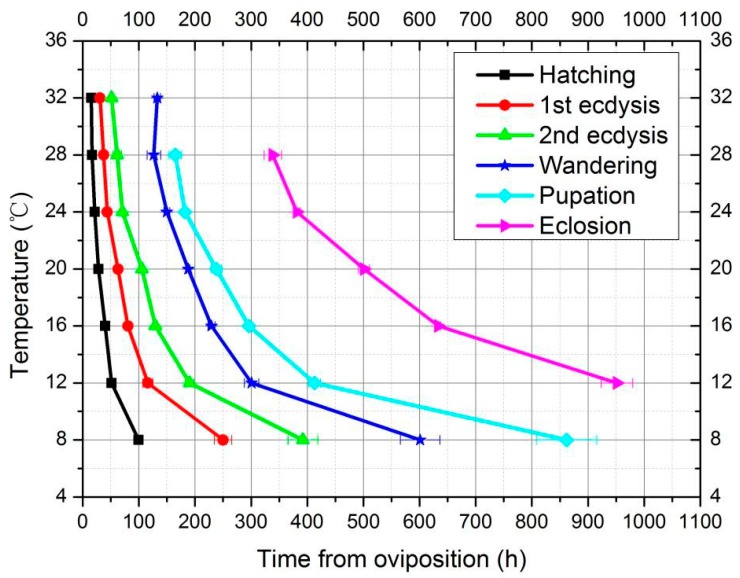
Isomorphen diagram of *A. grahami.* The duration (h) of each developmental event (hatching, first-ecdysis, second-ecdysis, wandering, pupation, and eclosion) plotted with the time from oviposition to the onset of each event. Each curve corresponds to a developmental event, and the error bar is the standard deviation of the time for each event.

The isomorphen diagram (Figure 3) was plotted based on the duration of different developmental events (x-axis) at different constant temperatures (y-axis). In the temperature range of 8–32 °C, the duration between each developmental event (hatching, first ecdysis, second ecdysis, pupation and eclosion) gradually decreased as the temperature increased, while only the specific time of the 3rd instar larvae and wandering were prolonged at 32 °C, and the curve displayed an distinct direction compared with the other six temperatures tested. With the temperature increasing, the distance between each curve was gradually shortened.

#### 3.2.2. Thermal Summation Model

**Figure 4:**
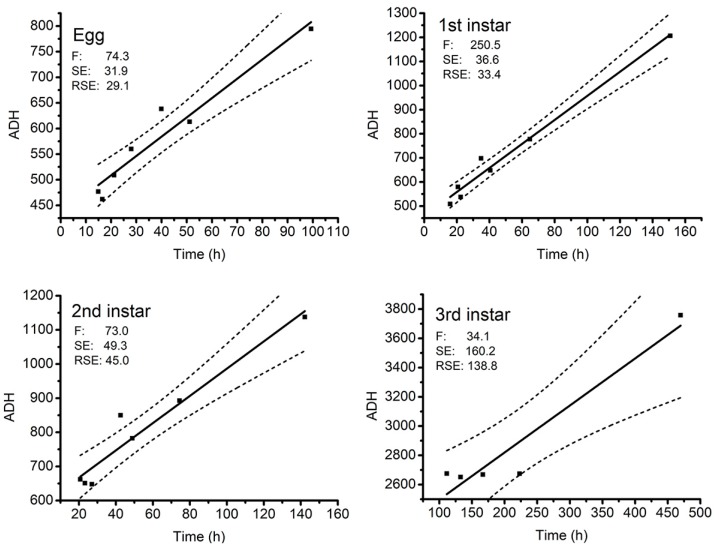

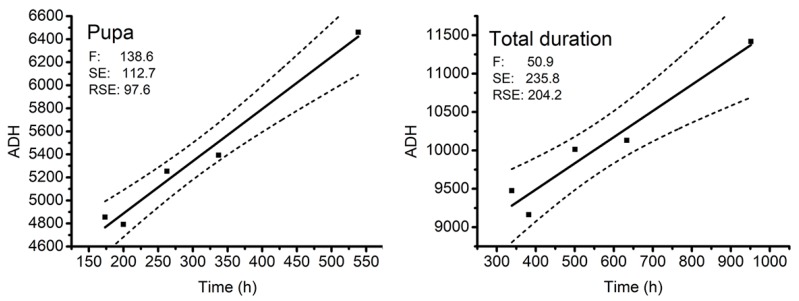
Thermal summation models of total developmental stages of *A. grahami*. represents the 95% confidence interval. F, SE, RSE respectively represent F statistic, standard error, residual standard error.

Six thermal summation models were established by linear regression analyses based on the duration of six developmental events and the entire developmental period as the x-axis and degree hours as the y-axis at each developmental stage (Figure 4). With the exception of the linear regression of 3rd instar larvae with a coefficient of determination (R^2^) value 0.89, the remaining five thermal summation models all had an R^2^ value above 0.92, indicating that the data of five models had a relatively good fit to linear models (Table 3). The data of 3rd instar larvae at 32 °C was poorly correlated with linear models. Hence, we rejected these data from the regression analysis. The developmental threshold temperature *D*_0_ and thermal summation constant K of *A. grahami* during the total developmental process calculated by the thermal summation model were 3.41 ± 0.48 °C and 8125.2 ± 288.4-degree hours, respectively (Table 3).

#### 3.2.3. Larval Body Length and Weight Changes over Time and Isomegalen Diagram

**Figure 5:**
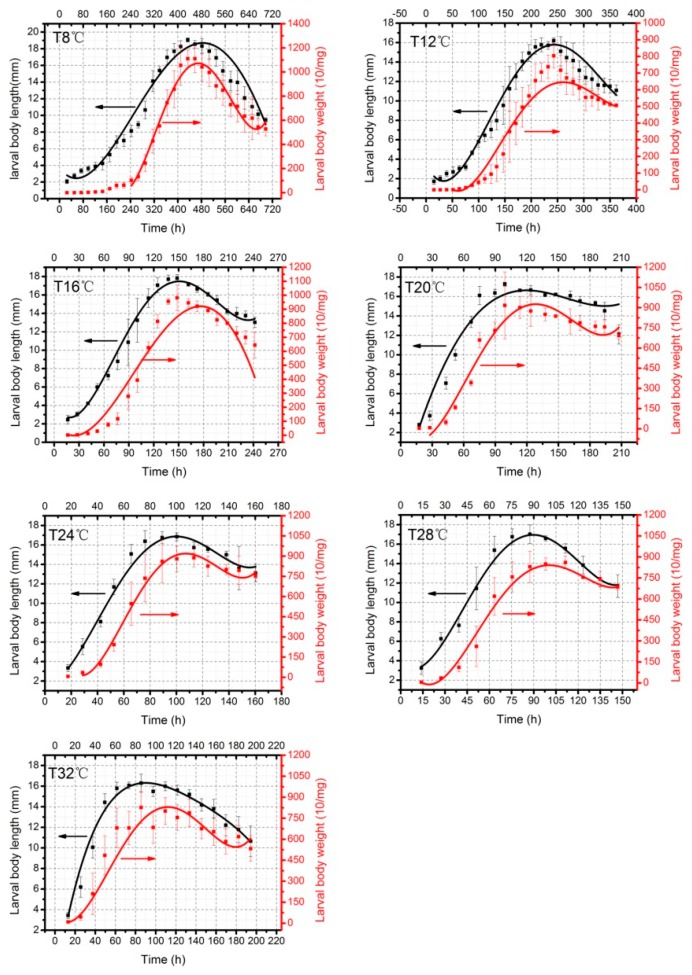
Changes in larval body length and weight of *A. grahami* at different constant temperatures. Larval body length changes were described in the left y-axis, and the larval body weight changes were shown in the right y-axis (10 larvae body weight as one unit).

Changes in larval body length and weight of *A. grahami* at different temperatures are shown in Figure 5; at each constant temperature the larval body length changes are described on the left y-axis, and the larval body weight changes are shown on the right y-axis (10 larvae body weight as a single unit). The equations in Table 4 show the changes in larval body length (*L*) with time (*T*), and the equations of changes in larval body weight (*W*) with time (*T*) described in Table 5. Both equations used time after hatching as the independent variable and larval body length/weight as the dependent variable. The R^2^, F value and P value all suggested that the fit of the equations was high.

**Figure 6:**
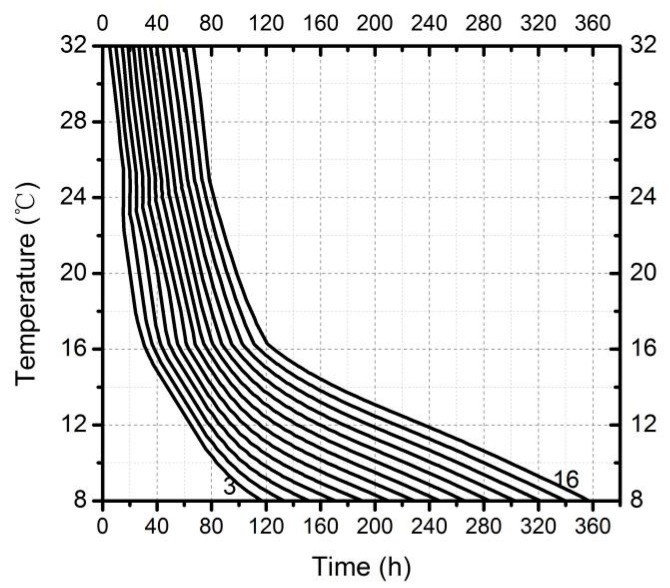
Isomegalen diagram of *A. grahami* larvae from hatching to peak feeding stage. Time was plotted against temperature where each line represents developmental larval length in 3–16 mm, size is indicated by the number at the lower margin of each contour.

The isomegalen diagram (Figure 6) was established by larval body length changes (changes in length only up to peak feeding stage, z-axis), time since oviposition (x-axis) and constant temperatures (y-axis); from left to right respectively represented larval body length changes from 3 mm to 16 mm. The distances between the contour lines became wide with decreasing temperature, suggesting that the larvae took a longer time to grow 1 mm.

## 4. Discussion

Fluctuating temperatures models were designed with monthly average minimum and monthly average maximum temperatures that occurred over the past 7 years in South Central China during winter and spring days, when temperature ranges of 6 °C can be experienced. In addition, we only put eggs into the climatic chamber in the morning, because we observed the peak oviposition of *A. grahami* concentrated at 7:00–12:00 am in our laboratory colony. Moreover, this was in accordance with bionomics and facilitated easy survival of the eggs in a relatively long period of warmer conditions [31].

Experimental results showed that the three groups of fluctuating temperatures had a longer developmental time and a slower developmental rate compared with the groups of constant temperatures, and these results were greatly in accordance with our fluctuating temperatures models. The time of the low-temperature segment (temperatures lower than the average temperature) in our model was too long at 13 h, and the high-temperature segment was only 11 h. We thought the impact on *A. grahami* at low-temperature was greater than at high-temperature. In the low-temperature periods, the *A. grahami* grew slowly, therefore, the groups of fluctuating temperatures developed in a slower manner. In addition, in the three groups of fluctuating temperatures vs. constant temperatures, the duration of developmental events became shorter with the increased temperature, and significance of difference vanished, which suggested that the average temperature of fluctuating temperatures was closer to the optimum temperature, and the difference between fluctuating temperatures and constant temperatures was more diminished in our models.

Only a few examples of forensic entomology research conducted rearing experiments under fluctuation conditions. In Dadour’s study [22], a comparison was made of the developmental rates between constant and cyclic temperatures in winter and summer temperature regimes, the results showed different developmental times of *Hydrotaea rostrate* from first stage larvae to emergent adult in constant and cyclic temperatures, *Hydrotaea rostrate* took more time for development in summer cyclic temperatures but took less time for development in winter cyclic temperatures, while statistical analysis were not significant. Clarkson compared the developmental rate of *Protophormia terraenovae* between under constant (20 °C) and fluctuating temperature (average temperature of naturally fluctuation = 19.7 °C) regimes [20]. The results showed the developmental rate under fluctuating temperatures was slower than that under constant temperatures in 1st, 2nd, and 3rd instar stages, with no difference in pupa. Likely, this result was caused by the lack of 0.3 °C in the average temperature of fluctuating temperatures. In 2010, Niederegger carried out similar research [15]. He found faster development under fluctuating temperatures for *Sarcophaga argyrostoma* and *Lucilia illustris* but slower development for *Calliphora vicina* and *Calliphora vomitoria*. His fluctuating temperature model was also designed by natural climate, but the span was too big (24 °C). In his model, the minimum temperature was close to the presumed *D*_0_ of *Calliphora vicina*, and *Lucilia illustris*, and the maximum temperature would possibly cause the death of *Calliphora vicina* larvae; this was against the standards of experimental design [32,33]. Moreover, the authors did not conduct a significant analysis. The latest paper was a study of the effect of fluctuating temperatures on the development of *Protophormia terraenovae* [21], with the same average temperature of two different ranges of fluctuating temperature compared with their mean constant temperatures. The results indicated development was fastest at the greater fluctuation and slowest at the constant temperatures, but showed similar percentages of developmental time at each stage. Interestingly, there were a transitory duration with the temperature under 10 °C (*Protophormia terraenovae* cannot complete development) in both fluctuating temperatures; the temperature most of the time was higher than the average temperature in this model, which may be the reason for the developmental rate of the fluctuating temperatures being faster than that of constant temperatures.

Insects in fluctuating temperatures research within the permissive thermal range can result in diverse responses [34], including accelerated development [35,36,37,38,39,40], delayed development [35,40,41], or unchanged in developmental rate [36]. One explanation for the various results in fluctuating temperatures studies on development may depend on the thermal mean that is used and its proximity to development thresholds [33,36], and the amplitude of fluctuating temperatures [33]. Different experiments on the effect of fluctuating temperatures had different results, which were highly dependent on a fluctuating temperatures model and the thermal tolerance of the insect. Moreover, fluctuating temperatures using deleterious (too high or too low) temperatures generally delay development compared with development at optimal constant temperatures [42,43]. This phenomenon is known as the Jensen’s inequality [44,45]. Although there have been reports that used developmental data of constant temperatures to successfully estimate PMI [46], many researchers have concluded that using constant temperatures is an unrealistic approach for studying the thermal responses of insects that typically live in thermally variable environments [33,34]. Besides, fluctuating temperatures should be incorporated into predictive models of growth.

There have only been a few studies conducted on *A. grahami* development [10,47,48,49,50]. Many factors can affect the development under constant temperatures, such as regional differences and rearing conditions (food, the control of temperature/humidity/photoperiod, the quantities/density of larvae, laboratory equipment and conditions, and the method of observation/sampling/preserved larvae). Our results in constant temperatures are strikingly similar to the data reported by Wang et al. [50]. The reason is that we use the same method to raise *A. grahami* and to conduct the experiments, moreover, we use the *A. grahami* is geographically close. The different that we use the pig lung to raise *A. grahami* when Wang use lean pork. In our *A. grahami* rearing process, we always kept *A. grahami* in the laboratory at 24 °C, 70% humidity and with natural light, and prevented other insects which are harmful for *A. grahami* development from getting into the rearing room. In our results, the pupa in F8 °C T8 °C and F12 °C failed to make eclosion, and the pupae were vacuous (Supplementary Materials Figure S3). This is different from Kikuo’s research [51], who conducted an experiment with the natural conditions of 2.0–7.0 °C and 7.0–13.0 °C. He found that both groups can make eclosion, although it is not clear why this is the case. However, in our experiment that the larvae had experienced a long period of cold in the wandering stage, and there was little stored energy to grow into an adult. Notably, we calculated the *D*_0_ of pupae was 4.54 ± 0.39 °C; this number was a predicted value and insect development under fluctuating acclimation regimes would have a greater tolerance of amplitude in temperature [39,52,53,54], when compared with development under constant acclimation regimes. In the results of constant temperatures, the durations of wandering in T28 °C were erratically prolonged; this may be explained by the thermal performance curves in Jensen’s inequality [45]; detrimental temperatures had an unnatural effect on biological behavior. The same reason may also explain the low eclosion rate (<10%) in T28 °C and the prolonged durations of 3rd instar larvae in T32 °C; after wandering the larvae cannot enter the pupal stage and the larvae become desiccated and die in T32 °C, indicating that 32 °C is close to the highest extreme temperature at which *A. grahami* cannot enter into the pupal stage, which is similar to Kikuo’s research [51], Kikuo found that *A. grahami* in the natural temperatures 28.0–34.5 °C hardly enter the pupal stage and failed to lead to eclosion. The above abnormal performance is a response to high temperature damage. In Kikuo research, he found *A. grahami* can’t hatch in natural temperatures 3.5–7.5 °C, which corresponds with our data about *D*_0_ of Egg 3.7 ± 0.43 °C.

From the graph of ADH (Figure 4), we can observe that the points were closer with increased temperature, and we rejected the data of 3rd instar in T28 °C and T32 °C, because they were responses to high temperature damage and were not developmental in nature. Remarkably, we tried to show the length and weight of larvae in the same diagram in 8–32 °C, while aiming to rapidly determine the age of larvae when we collected them.

Commonly, forensic entomologists usually use three models to estimate PMI. Isomorphen diagrams are the most basic model, with the duration of development events as the X-axis and temperature as the Y-axis. This model is simple to use and easily explained in court, however the simplicity of this model compromises the accuracy of the output [3,55]. Isomegalen diagrams provide more accurate PMI estimates than isomorphen diagrams [3,56]. The first step is to judge whether the larvae were in the feeding stage or the wandering stage when using this model, as this model only establishes the changes in length from hatching to peak feeding, but is not applicable when larvae shrink naturally [3,46,55]. Thermal summation models are the most sophisticated of the three common models and are the preferred model to use when estimating PMI [1,3]. However, a criticism of this model is the high variance in the upper and lower developmental temperature extremes [3].

The three models could be used in most forensic investigation, but with regard to *A. grahami*, some particular conditions, such as a cold climate with the temperature close to *D*_0_, or low ambient temperature with rainfall, are not suited to these models. Finally, we suggest that when designing the temperatures for collecting the developmental data of forensically important flies, the bionomics of flies and the climate conditions that these flies are active in should be taken into consideration. Further studies are needed to rear and observe specimens in the same laboratory conditions using similar methodology.

## 5. Conclusions

This study provides the developmental data of *A. grahami* in fluctuating and constant temperatures for determining the post-colonization interval and play an important role in the estimation of the PMI_min_. Our results indicated that the developmental rate in fluctuating temperatures was slower than in constant temperatures over the whole developmental cycle, and most developmental durations had significant differences between fluctuating temperatures and constant temperatures. It is unrealistic to only use developmental data in constant temperatures to estimate the PMI_min_, especially in extreme environments. Our developmental data including fluctuating temperatures provides an important reference value for the use of *A. grahami* in estimating the PMI_min_ in cold weather.

## Figures and Tables

**Table 1:** Mean (±SD) developmental duration of *A.* *grahami* at constant and fluctuating temperatures.

Developmental Stages (h)							
Temperature (°C)	Egg	First-Instar	Second-Instar	Third-Instar	Wandering	Pupa	Total Duration
8	99.3 ± 4.3 ^a^	150.8 ± 13.5 ^a^	142.2 ± 18.3 ^a^	208.6 ± 20.1 ^a^	261.2 ± 20.7 ^a^	X	
6–12	133.9 ± 16.1 ^b^	166.8 ± 3.6 ^b^	155.6 ± 2.9 ^b^	253.0 ± 20.2 ^b^	354.6 ± 15.4 ^b^	X	
12	51.1 ± 4.0 ^c^	64.8 ± 5.9 ^c^	74.4 ± 5.0 ^c^	110.5 ± 11.7 ^c^	112.4 ± 11.3 ^c^	538.4 ± 22.1	951.6 ± 28.3
10–16	56.7 ± 3.8 ^cd^	74.2 ± 5.2 ^d^	90.5 ± 7.1 ^d^	142.0 ± 10.3 ^d^	133.2 ± 7.9 ^d^	X	
16	39.9 ± 2.5 ^e^	40.5 ± 3.1 ^e^	48.9 ± 1.2 ^e^	100.2 ± 7. 3^e^	66.6 ± 6.5 ^e^	337.1 ± 5.1 ^e^	633.2 ± 3.6 ^e^
14–20	44.0 ± 1.6 ^e,f^	46.8 ± 1.5 ^ef^	55.3 ± 3.7 ^ef^	110.4 ± 7.4 ^ef^	87.0 ± 3.3 ^f^	389.1 ± 10.4 ^f^	732.6 ±14.0 ^f^
20	28.0 ± 1.3	34.9 ± 2.1	42.5 ± 3.5	82.5 ± 3.3	50.1 ± 6.1	262.7 ± 3.9	500.7 ± 9.8
24	21.2 ± 0.9	22.4 ± 0.4	27.0 ± 1.7	79.2 ± 4.2	32.3 ± 3.7	199.7 ± 5.1	381.8 ± 5.1
28	16.5 ± 1.6	20.7 ± 3.7	23.25 ± 4.6	65.4 ± 7.2	38.4 ± 4.6	173.4 ± 12.2 ^▲^	338.4 ± 15.5
32	14.9 ± 0.7	15.9 ± 0.9	20.7 ± 2.2	81.3 ± 4.5	83.2 ± 4.9 *	X	
36	X	X	X	X	X	X	

X represent failure enter the developmental stages. * represent the interval from wandering to larvae wizened and died in 32 °C. ▲ represents results where the eclosion rate was very low (<10%). ^a–f^ represent Values within the same column followed by the same letter do not differ significantly from each other based on a one-way ANOVA + LSD test at *p* < 0.05.

**Table 2:** Curve fitting equation, degrees of freedom (df), and coefficients of determination (R^2^) of the relationship between the body length (*L*) (mm) of *A. grahami* larvae and the time after hatching (*T*) (h) at three fluctuating temperatures 6–12 °C (F8), 10–16 °C (F12 °C), 14–20 °C (F16 °C), and constant temperatures 8 °C (T8 °C), 12 °C (T12°C), 16 °C (T16 °C).

Temperature (°C)	Equation	df	R^2^
F8	*L* = 3.567 − 0.048*T* + 4.0 × 10^−4^*T*^2^ − 6.8 × 10^−7^*T*^3^ + 3.3 × 10^−10^*T*^4^	34	0.991
T8	*L* = 3.650 − 0.044*T* + 4.6 × 10^−4^*T*^2^ − 7.8 × 10^−7^*T*^3^ + 3.4 × 10^−10^*T*^4^	24	0.976
F12	*L* = 2.449 − 0.046*T* + 9.9 × 10^−4^*T*^2^ − 2.9 × 10^−6^*T*^3^ + 2.0 × 10^−9^*T*^4^	29	0.989
T12	*L* = 3.498 − 0.112*T* + 2.1 × 10^−3^*T*^2^ − 7.8 × 10^−6^*T*^3^ + 8.6 × 10^−9^*T*^4^	25	0.990
F16	*L* = 3.134 − 0.130*T* + 3.5 × 10^−3^*T*^2^ − 1.8 × 10^−5^*T*^3^ + 2.7 × 10^−8^*T*^4^	20	0.991
T16	*L* = 4.149 − 0.168*T* + 5.4 × 10^−3^*T*^2^ − 3.5 × 10^−5^*T*^3^ + 6.4 × 10^−8^*T*^4^	15	0.998

**Table 3:** Mean (±SE) of developmental threshold temperatures (*D*_0_) and thermal summation constants (*K*) for six developmental stages and the total developmental period of *A. grahami*, and the coefficient of determination (R^2^) of thermal summation models.

Developmental Stages	*K* (Degree Hours)		*D*_0_ (°C)		R^2^
	Mean	SE	Mean	SE	
Egg	433.2	20.8	3.77	0.43	0.92
First instar	458.6	21.0	4.99	0.32	0.98
Second instar	587.4	31.4	3.99	0.47	0.92
Third instar	2176.5	141.1	3.21	0.55	0.89
Pupa	3980.4	126.9	4.54	0.39	0.97
Total duration	8125.2	288.4	3.41	0.48	0.93

**Table 4:** Curve fitting equation, degrees of freedom (df), and coefficients of determination (R^2^) of the relationship between the body length (*L*) (mm) of *A. grahami* larvae and the time after hatching (*T*) (d) at seven constant temperatures.

Temperature (°C)	Equation	df	R^2^
8	*L* = 3.650 − 0.044T + 4.6 × 10^−4^T^2^ − 7.8 × 10^−7^T^3^+3.4 × 10^−10^T^4^	24	0.976
12	*L* = 3.498 − 0.112T + 2.1 × 10^−3^T^2^ − 7.8 × 10^−6^T^3^+8.6 × 10^−9^T^4^	25	0.990
16	*L* = 4.149 − 0.168T + 5.4 × 10^−3^T^2^ − 3.5 × 10^−5^T^3^+6.4 × 10^−8^T^4^	15	0.998
20	*L* = −3.777 + 0.395T – 2.2 × 10^−3^T^2^ + 1.3 × 10^−6^T^3^+1.0 × 10^−8^T^4^	12	0.979
24	*L* = 1.550 + 0.0024T – 6.7 × 10^−3^T^2^ − 7.4 × 10^−5^T^3^+2.2 × 10^−7^T^4^	8	0.989
28	*L* = 3.983 − 0.189T + 1.2 × 10^−2^T^2^ − 1.3 × 10^−4^T^3^+4.1 × 10^−7^T^4^	7	0.990
32	*L* = −3.340 + 0.591T – 6.3 × 10^−3^T^2^ + 2.8 × 10^−5^T^3^−4.8 × 10^−8^T^4^	11	0.990

**Table 5:** Curve fitting equation, degrees of freedom (df), and coefficients of determination (R^2^) of the relationship between the body weight (mg) (10/mg) of *A. grahami* larvae and the time after hatching (*T*) (d) at seven constant temperatures.

Temperature (°C)	Equation	df	R^2^	Errors as Weight
8	W = 6858.957 − 82.870T + 0.342T^2^ − 5.5 × 10^−4^T^3^ + 3.1 × 10^−7^T^4^	15	0.966	no weighting
12	W = 359.021 − 13.670T + 0.156T^2^ − 5.3 × 10^−4^T^3^ + 5.7 × 10^−7^T^4^	22	0.993	no weighting
16	W = 86.127 − 7.780T + 0.191T^2^ − 8.0 × 10^−4^T^3^ + 7.1 × 10^−7^T^4^	15	0.988	instrumental
20	W = 35.782 − 15.203T + 0.549T^2^ − 4.1 × 10^−3^T^3^ + 9.1 × 10^−6^T^4^	11	0.939	no weighting
24	W = 703.987 − 58.104T + 1.516T^2^ − 1.2 × 10^−2^T^3^ + 3.3 × 10^−5^T^4^	7	0.981	no weighting
28	W = 260.311 − 31.548T + 1.089T^2^ − 0.01T^3^ + 2.9 × 10^−5^T^4^	7	0.998	instrumental
32	W = 63.503 − 9.915T + 0.490T^2^ − 4.2 × 10^−3^T^3^ + 1.0 × 10^−5^T^4^	11	0.980	instrumental

## Supplementary Materials

The following are available online at https://www.mdpi.com/2075-4450/10/7/184/s1. Figure S1: Change in larval body weight with time was shown in three groups constant versus fluctuating temperatures. The curve represents the fitting of larval body weight and time; Figure S2: In T8 °C and F12 °C adults were failing to make eclosion, and the pupa were vacuous. Table S1: Curve fitting equation, degrees of freedom (df), and coefficients of determination (R^2^) of the relationship between the body weight (W) (mg) of *A. grahami* larvae and the time after hatching (T) (h) at three fluctuating temperatures 6–12 °C (F8 °C), 10–16 °C (F12 °C), 14–20 °C (F16 °C), and constant temperatures 8 °C (T8 °C), 12 °C (T12 °C), 16 °C (T16 °C).
