# Supplementary material for: Impact of Constant Versus Fluctuating Temperatures on the Development and Life History Parameters of Aldrichina grahami (Diptera: Calliphoridae)
Source: Insects. 2019 Jun 26;10(7):184. doi: 10.3390/insects10070184 (PMC6681350; doi:10.3390/insects10070184)
Supplement: Supplementary file 1 [file insects-10-00184-s001.pdf]

insects

Article

Impact of Constant Versus Fluctuating Temperatures on the Development and Life History Parameters of *Aldrichina grahami* (Diptera: Calliphoridae)

Wei Chen [1,†], Li Yang [1,†], Lipin Ren [1], Yanjie Shang [1], Shiwen Wang [2] and Yadong Guo [1,*]

[1] Department of Forensic Science, School of Basic Medical Sciences, Central South University, Changsha 410013, China; chen_wei93@126.com (W.C.); yangli1015@csu.edu.cn (L.Y.); renlp87@126.com (L.R.); shangyj@csu.edu.cn (Y.S.)
[2] Department of Forensic Science, School of Basic Medical Sciences, Xinjiang Medical University, Urumqi 830011, China; wsw180325@126.com
* Correspondence: gdy82@126.com
† These two authors contributed equally to this work.

Received: 7 May 2019; Accepted: 18 June 2019; Published: date

Supplementary Materials

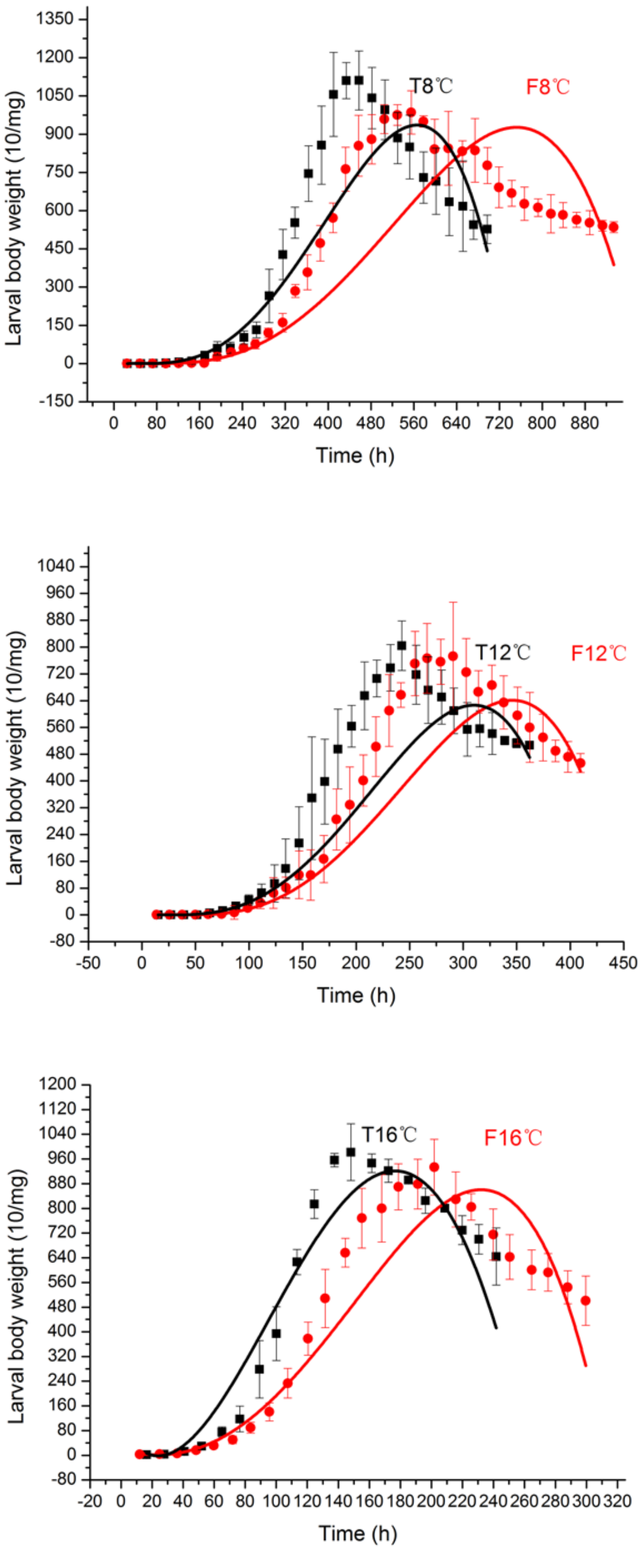

Figure S1. Change in larval body weight with time was shown in three groups constant versus fluctuating temperatures. The curve represents the fitting of larval body weight and time.

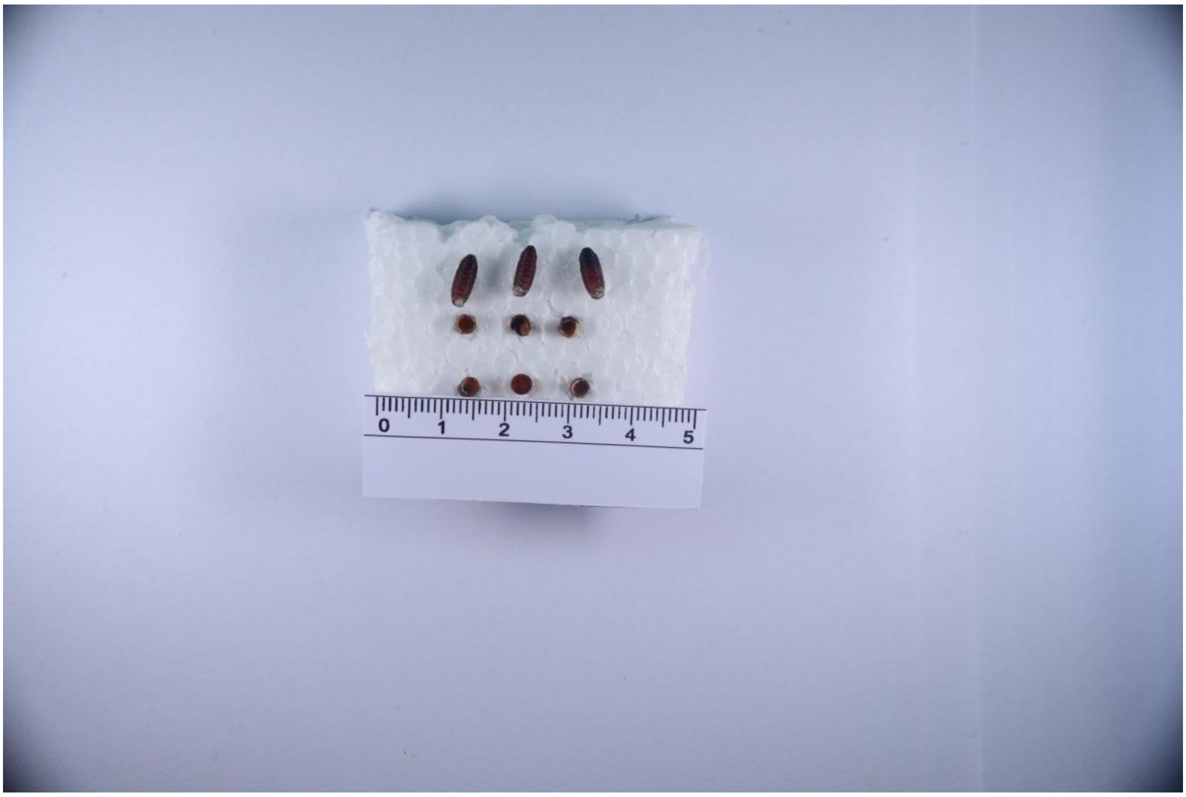

Figure S2. In T8 °C and F12 °C adults were failing to make eclosion, and the pupa were vacuous.

Table 1. Curve fitting equation, degrees of freedom (df), and coefficients of determination (R^2) of the relationship between the body weight (W) (mg) of *A. grahami* larvae and the time after hatching (T) (h) at three fluctuating temperatures 6–12 °C (F8 °C), 10–16 °C (F12 °C), 14–20 °C (F16 °C), and constant temperatures 8 °C (T8 °C), 12 °C (T12 °C), 16 °C (T16 °C).

Temperature (°C)	Equation	df	R^2
F8	$w = -1.837 + 0.139T - 0.00252T^2 + 1.5E{-}5T^3 - 1.3E{-}8T^4$	34	0.863
T8	$w = -2.348 + 0.197T - 0.00445T^2 + 3.5E{-}5T^3 - 3.9E{-}8T^4$	24	0.879
F12	$w = -2.248 + 0.331T - 0.01082T^2 + 1.2E{-}4T^3 - 2.1E{-}7T^4$	29	0.864
T12	$w = -1.320 + 0.189T - 0.00847T^2 + 1.3E{-}4T^3 - 2.8E{-}7T^4$	25	0.970
F16	$w = 15.269 - 1.116T - 0.01907T^2 + 1.7E{-}4T^3 - 7.0E{-}7T^4$	20	0.942
T16	$w = 86.127 - 7.780T - 0.019058T^2 - 8.0E{-}4T^3 - 7.0E{-}7T^4$	15	0.988
